# Studies on the human choroid plexus *in vitro*

**DOI:** 10.1186/2045-8118-10-10

**Published:** 2013-02-07

**Authors:** Zoran B Redzic

**Affiliations:** 1 Department of Physiology, Faculty of Medicine, Kuwait University, Safat, 13110, Kuwait

**Keywords:** Human choroid plexus, Cerebrospinal fluid, Blood-cerebrospinal fluid barrier, Choroid plexus papilloma, Choroid plexus carcinoma, Primary culture, Choroid plexus epithelium

## Abstract

The role of human choroid plexus (CP) epithelium in the transport of solutes between the blood and the cerebrospinal fluid and/or in secretion processes may be studied by employing several experimental approaches. There are a number of *in vitro* techniques for human CP epithelium (CPE) and all have limitations that do not exclude them *a priori*, but that should be carefully taken into consideration. Developmental and morphological studies have been largely performed on human choroid plexus samples of either embryonic or post-mortem origin. Functional uptake studies may be performed on pathologically unaltered CP samples obtained during surgical removal of choroid plexus tumors. This approach can be used to explore transport processes mainly across the apical side of the CPE, but cannot be used to study vectorial transport across the CPE. Also, these samples have limited viability. A monolayer of CPE in culture, grown on permeable supports, provides the best available tool to study transport processes or polarized secretion by the CP, but thus far only limited attempts to culture these cells have been published and they mainly include data from neoplastic CPE. A study that used a human papilloma-derived cell line in culture showed that it forms a monolayer with barrier properties, although the cells express pleomorphic and neoplastic features and lack contact inhibition. Other cell cultures express some CPE markers but do not develop tight junctions/barrier properties. This article reviews the main characteristics and limitations of available *in vitro* methods to study human CPE, which could help researchers choose an appropriate experimental approach for a particular study.

## Review

### Introduction

A constant and well-controlled composition of extracellular fluid in the central nervous system (CNS) is essential for neuronal processing. Thus, all existing vertebrates have cellular structures that provide efficient physical separation of the circulating plasma from brain extracellular fluids [1]. The two most important are the endothelial blood–brain barrier (BBB) which separates plasma from the interstitial fluid of the brain, and the epithelial blood-cerebrospinal fluid barrier (BCSFB), which separates blood from ventricular cerebrospinal fluid (CSF). These cellular structures impede free paracellular diffusion of hydrophilic solutes and transcellular diffusion of lipophilic compounds from circulating plasma into extracellular fluids of the brain and exclude xenobiotics, providing the controlled environment required for optimal CNS function.

The BCSFB is formed by the epithelium of the choroid plexuses (CPs). The BCSFB has a considerable surface area for exchange between the blood and the CSF through the presence of microvilli on the apical surface and interdigitations on the basolateral surface. The barrier phenotype of this cellular interface is achieved mainly by continuous tight junctions (TJs) between adjacent cells of the CP epithelium (CPE). These intercellular structures greatly limit paracellular diffusion and, thus, exchange of polar solutes between the blood and the CSF [2]. Claudins 1, 2, 3 and 11 are the most important members of the claudin family of TJ proteins of the CPE [3]. Claudin 11 in the TJs of the CPE is responsible for parallel-stranded TJs, observed in freeze-fracture morphology [3,4]. This relationship between structure and molecular composition of the TJs is important and could be used as an indicator of whether or not the CPE maintains functional features *in vitro*. Together with restriction of free paracellular diffusion, the presence of a large number of transport systems and intracellular metabolic activities contribute significantly to the barrier properties of the BCSFB [5]. Many transport systems and ion transporters and channels have an unequal distribution between the basolateral membrane, that faces CP interstitial fluid, and the apical membrane, that faces ventricular CSF. These polarized transport and secretion processes in the CPE are essential for secretion of ventricular CSF.

Several experimental approaches have been used to explore functions of the human CPs. Human CSF samples are frequently taken for diagnostic purposes and are used mainly for detection of biomarkers for developmental disorders or for various CNS pathologies, including neuroinflammatory and neurodegenerative diseases [6,7]. However, it is difficult to explore functions of the human CPE by analysis of blood plasma and CSF samples because the CSF is constantly renewed *in vivo* and diffusion of solutes from the brain interstitial fluid into the CSF influences the CSF composition.

The most accessible human CP samples were those that were either taken from aborted embryos or from post-mortem adult brains. Human embryonic CP samples have been used to analyze the expression of transport proteins that are important for the CP function, for instance, ATP-binding cassette transporters ABCB1, ABCB4, ABCC1 [8], or to explore developmental changes in CP morphology and protein expression [9]. Samples taken post-mortem have been used to explore expression of various proteins in the human CP; for example, the expression of hepatocyte nuclear factor 4 (HNF4-alpha), a transcription factor that targets numerous drug-metabolizing enzymes and drug transporters important for the detoxifying function of the CPs, has been explored in detail in CP samples from adult brains [10]. Although post-mortem samples could not be used for functional studies, mapping protein expression in human CP taken post-mortem can be used to predict function. For example, it has been shown that the water channel, aquaporin-1, Na^+^-K^+^-ATPase ***α***1-subunit and Na^+^-K^+^-2Cl^−^ cotransporter are localized apically in the human choroid plexus epithelial cells; the Cl^−^/HCO_3_ ^−^ exchanger, AE2, is localized basolaterally, as is the Na^+^-dependent Cl^−^/HCO_3_ ^−^ exchanger, NCBE, and the electroneutral Na^+^-HCO_3_ ^−^ cotransporter, NBCn1. No immunoreactivity was found for the Na^+^-dependent acid/base transporters NHE1 or NBCe2 [11]. Since this pattern of distribution is very similar to that observed in other species, such as rat or mouse, authors have concluded that the preserved pattern of expression across species suggests central roles for these transporters in CSF production [11]. The main limitation of this approach is that the CP samples can only be collected after several hours have elapsed and may be taken more than 1 day post-mortem; this delay in tissue processing could affect protein content in the CPE due to proteolysis [12]. An alternative approach would be to obtain human CP samples after neurosurgery and to use them immediately for molecular biology or for functional uptake studies or to produce a cell culture of human CPE. These approaches have several advantages and limitations that will be briefly reviewed in this article.

### Functional studies on human CP samples

Human CP samples may be obtained as spare material from neurosurgery. In most cases, a small amount of healthy CP tissue has to be dissected during surgical removal of a large choroid plexus papilloma. An important first step is to examine samples in order to separate pathologically-unaltered tissue which can be used either for uptake studies, immunocytochemistry or for molecular biology. Uptake studies can be performed with two radiotracers: a test molecule and a reference molecule which serves as an extracellular space marker. The latter is usually radiolabelled mannitol or sucrose or a larger extracellular space marker like inulin, the choice depending on the size and physical properties of the test molecule. The total amount of test molecule that is found in the tissue sample after the incubation consists of two different pools: a) test molecules that diffuse from aCSF into the CP extracellular fluid (ECF) and remain in the ECF; b) test molecules that entered the CPE during the course of the experiment, a process mediated by some of the equilibrative or concentrative transporters at the plasma membranes of the CP cells. It is not possible to estimate amounts of a test molecule in these two pools directly. However, the reference molecule, if properly selected, behaves similarly to the test molecule during the course of experiment, with the only difference being that it cannot be taken up by the cells, because of the absence of specific transport systems to mediate cellular entry. Thus, the amount of the test molecule in the ECF can be estimated by determining the reference molecule radioactivity / mg tissue protein. More accurate data are obtained if the reference molecule radioactivity is multiplied by the reference molecule DPM / test molecule DPM ratio in the sample. This approach was used to characterize nucleoside transport across the apical side of human CPE [13].

However, there are three factors that should be taken into account when considering this experimental technique. First, the basolateral (CP interstitial fluid)-facing side of the CPE is not easily accessible during these experiments because under the experimental conditions, no perfusion pressure is present in the microcirculation, a situation which could cause CP capillaries to collapse. Bearing in mind the histological structure of the CP [14], the diffusion of solutes from the aCSF into the CP interstitial fluid under these circumstances would be rather limited. Furthermore, the existence of TJs between epithelial cells of the CP occludes the paracellular space and further impedes diffusion of solutes from the aCSF into the CP interstitial fluid that surrounds the basolateral membrane [14]. Taking into account the importance of vectorial transport across the CPE, data obtained by this type of study have limited applicability. Second, the prospective duration of a single experiment is constrained by the limited viability of isolated CP samples. Third, a contribution of other cell types to the experimental data (e.g. to the rate of uptake or to the amount of mRNA or protein) cannot be excluded.

### In vitro cell cultures of human CPE

In order to study vectorial transport of solutes across the CPE or CSF secretion, samples of human CP could be used to produce an *in vitro* cellular model of the BCSFB. Several attempts have been made to produce cell cultures using either fetal CP or CP tumors. Samples of human CPs or CP tumors were used either as tissue explants, which were sources of epithelial cells with preserved viability, or to obtain single epithelial cells and epithelial cell clusters after the digestion with dispase, pronase or diluted trypsin (for more details on digestion of CP samples see [15]). Cells are then plated onto transwell permeable supports or into tissue culture plates.

Early attempts to culture human CPE date from 1949, when the first report of fetal human CP cells in tissue culture was published [16]. This early study used CPs from human fetuses of different gestational ages (crown-rump length measuring from 52–160 mm) and maintained them as tissue explants in rolling test tubes in a nutrient medium consisting of Tyrode solution, Locke-Lewis solution, human placental serum and chick embryo extract [16]. Epithelial cells migrated from these explants, forming islands (“plates”) or ribbons of cells that curved and branched [16]. An attempt was also made to culture adult brain cells, including CPE, from brain tissue that was taken either during biopsy or 6-24 h post-mortem; this tissue was maintained either as explants or was digested to single cells that were plated into culture plates [17]. Under these conditions, monolayers of CPE were established in less than one week and CPE growth was faster than from brain parenchyma [17]. CP cells from explants grew as bipolar spindle-shaped cells in an orderly fashion [18]. This culture was also used to explore the mechanism of cytomegalovirus infection and growth [19].

However, use of primary human CPE cell culture with either an embryonic or post-mortem/biopsy origin was gradually abandoned over the time. Possibly, the main reason for this was difficulty in obtaining well-preserved samples frequently enough to produce sufficient material for primary cultures. Also, tissue samples often produce a rather limited amount of primary cells with variability between different cultures that is often large, and which could be due to the variable times needed to obtain the tissue post-mortem. To overcome this problem, several cell lines were generated from neoplastic human CPE.

#### Human choroid plexus papilloma cell line (HIBCPP)

A choroid plexus papilloma is a tumor of a CPE origin occurring in cerebral ventricles, which is infrequently malignant [20]. Using malignant papilloma from a 29-year old woman, a research group in Japan established a malignant cell line [21]. This was achieved by the digestion of the tumor tissue in dispase, followed by incubation of the digest at 37°C in 5% CO_2_ and air on uncoated and untreated cell culture plastics in Ham’s F-12 medium supplemented with 10% fetal calf serum (FCS) and antibiotics [21]. It was initially reported that these cells demonstrated pleomorphic and neoplastic features and lacked contact inhibition [21]. Thus, they formed heterogeneous multilayers [21]. For these reasons it was believed that this cell line was unlikely to be suitable for use as an *in vitro* model of the BCSFB [15].

**Figure 1:**
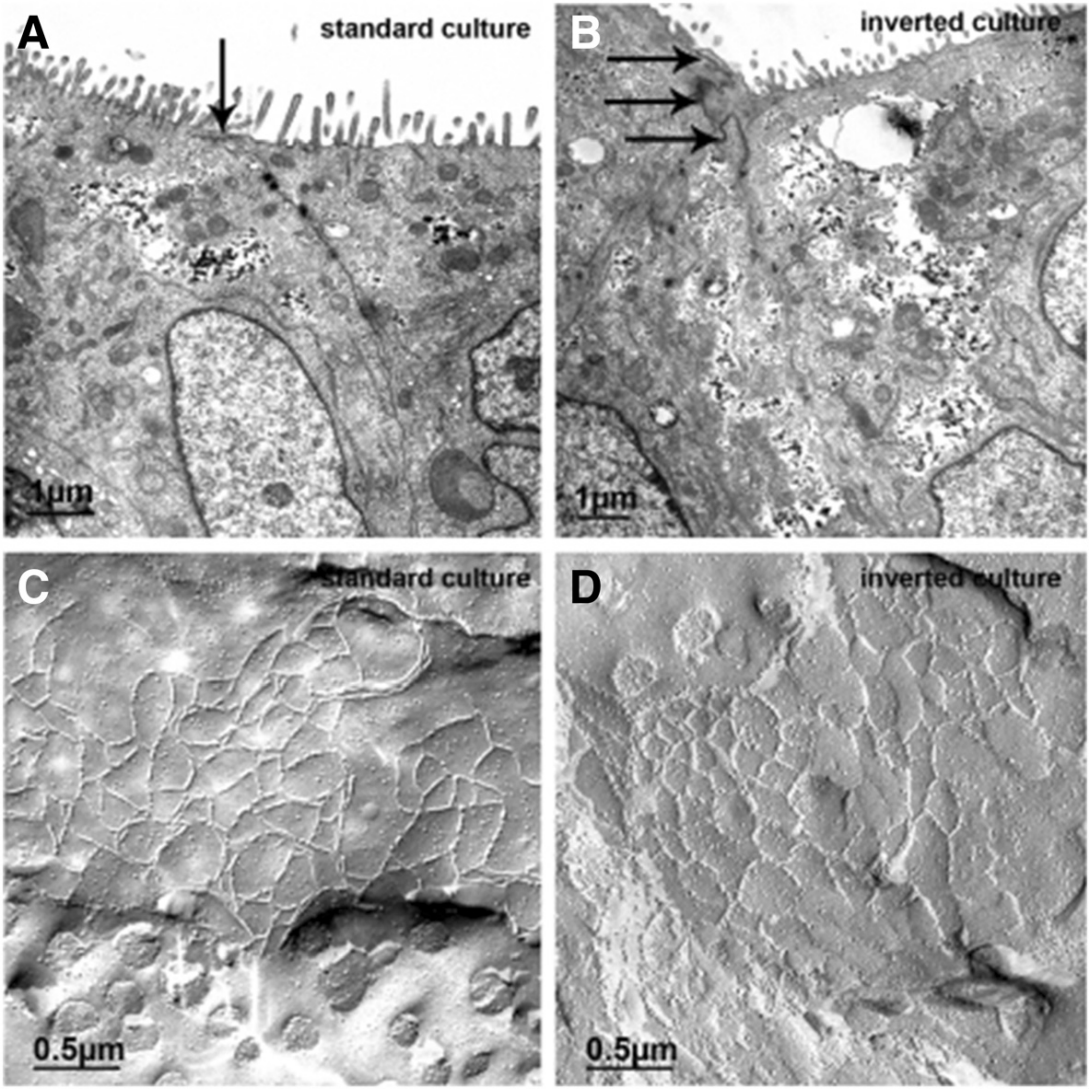
**Tight junctions (TJ) in human malignant choroid plexus papilloma cell line (HIBCPP) grown on transwell filter supports in the standard (A, C) and the inverted (B, D) culture system, respectively. **Transmission electron microscopy (**A**, **B**) shows that in both culture systems the cells are connected by TJs (arrows), which are located close to the apical side as indicated by the presence of microvilli. Examination of HIBCPP by freeze fracture electron microscopy (**C**, **D**) revealed a broad band of closely meshed TJ strands. The diameter of mesh was in the magnitude of 0.2 to 0.4 μm. Reproduced with permission from the author [22].

However, a recent report [22] has revealed that, if growth conditions are carefully optimized, with seeding density adjustment and selective trypsinization, these cells can develop some features of the CPE *in situ*, including increase in transepithelial electrical resistance [TEER] up to 500 Ω cm^2^, low paracellular diffusion of a paracellular marker FITC-labelled inulin, formation of the TJs between adjacent cells that were located close to the apical border (Figure 1) and the presence of microvilli [22].

**Figure 2:**
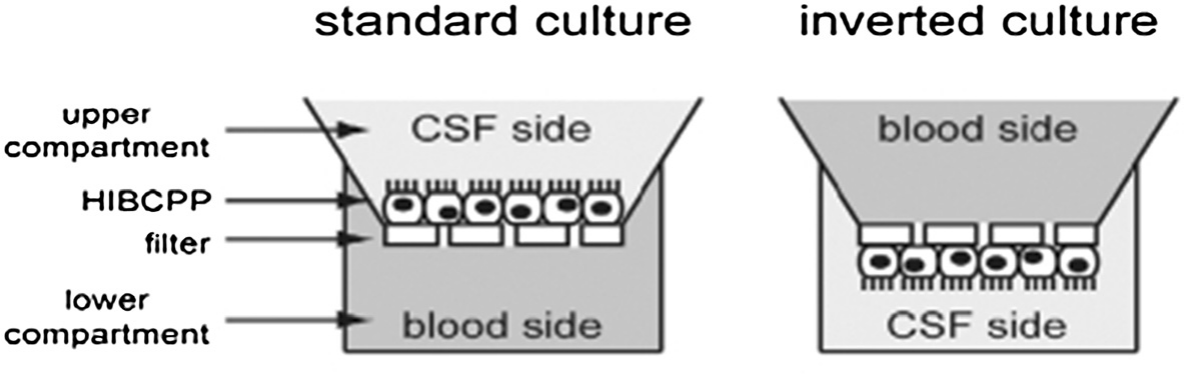
**Schematic representation of standard and inverted transwell filter systems. **Most studies that explored vectorial processes across the CPE have used a standard transwell filter system. The inverted system was originally developed to analyze bacterial invasion and translocation in a porcine CPE in culture [23] and it enabled studies of bacterial interaction with the basolateral (blood) side of the CPE. Reproduced with permission from the author [22].

To achieve these characteristics, HIBCPP cells were cultured in DMEM/HAM’s F12 medium that was supplemented with 15% FCS, L-glutamine and insulin and were seeded on transwell filters with 3.0 μm pore size, although 0.45 μm pore size could also be used [22]. HIBCPP cells were introduced either as the standard transwell filter system, with cells seeded on the upper side of the filter, or as the inverted transwell filter system, with cells seeded on the lower side of the filter (with the apical side of the cells facing the bottom of the well, Figure 2), the latter system being originally developed to study bacterial invasion across the CPE *in vitro*[23] and has also been used recently to study the permissiveness of human CPE for Echovirus 30 and T cell migration across the CPE layer [24].

HIBCPP cells reached optical confluence 3–4 days after seeding. At this point, cell density was ≈1.2 million cells / cm^2^ and the TEER started to rise, reaching several hundred Ω cm^2^ after a week [22]. This could be a consequence of the formation of TJs between adjacent cells, resembling the *in situ* structure of the CPE (Figure 1). However, as shown in Figure 1C and D, TJs displayed densely meshed strands, rather than a parallel-stranded appearance, which could indicate absence or dislocation of claudin 11. The TEER value in this culture appears to be strongly related to the amount of FCS in the medium from day 3–4 after seeding onwards, with cultures that were incubated with less FCS having higher TEERs. These cells expressed several proteins at the transcript level that are typical for the CPE, such as transthyretin and insulin-like growth factor 2.

However, the characteristics of this culture should be carefully considered for every study. First, by being derived from anaplastic CPE, this cell line is likely to show considerable differences in protein expression and cellular metabolism, when compared to pathologically-unaltered human CPE *in vivo*. Second, HIBCPP cells were used after more than 30 passages, a fact that is likely to further contribute to dedifferentiation of these cells. Third, their morphology appears to be rather different from the morphology of the CPE primary cultures: cells differed in size and did not display a typical “cobblestone-like” appearance of the CPE in primary cultures. Also, these cells possessed very large nuclei that in some cases almost filled the cells [22]. Fourth, HIBCPP cells often do not show contact inhibition and, thus, have a tendency to grow in multiple layers [21]. Thus, as described above, a careful adjustment of seeding density and selective trypsinization has to be applied in order obtain a cellular monolayer. Fifth, these cells change both doubling time and plating efficiency with increasing passages [21]. However, if all these factors are taken fully into consideration, this cell culture could be used as a proper tool, similar to the case of studies that explored bacterial and translocation and T cells migration across the human CPE [22,24].

There was another reported attempt to cultivate cells derived from a fragment of a fourth-ventricle CP papilloma; these were shown to form a monolayer with a pavement-like appearance and displayed ultrastructural features similar to those of the papilloma epithelial cells [25]. However, no further data could be found about this cell culture.

#### Human choroid plexus carcinoma cell line

Another human CPE cell line, CPC-2, was initially produced from a CPE carcinoma taken from a 2-month old boy, which is a rare tumor [26]. The tissue was mechanically dissociated and enzymatically digested after neurosurgery, then passed through a 70 μm strainer and cultured in Dulbecco’s minimum essential medium supplemented with 20% FCS under conventional culture conditions [26].

**Figure 3:**
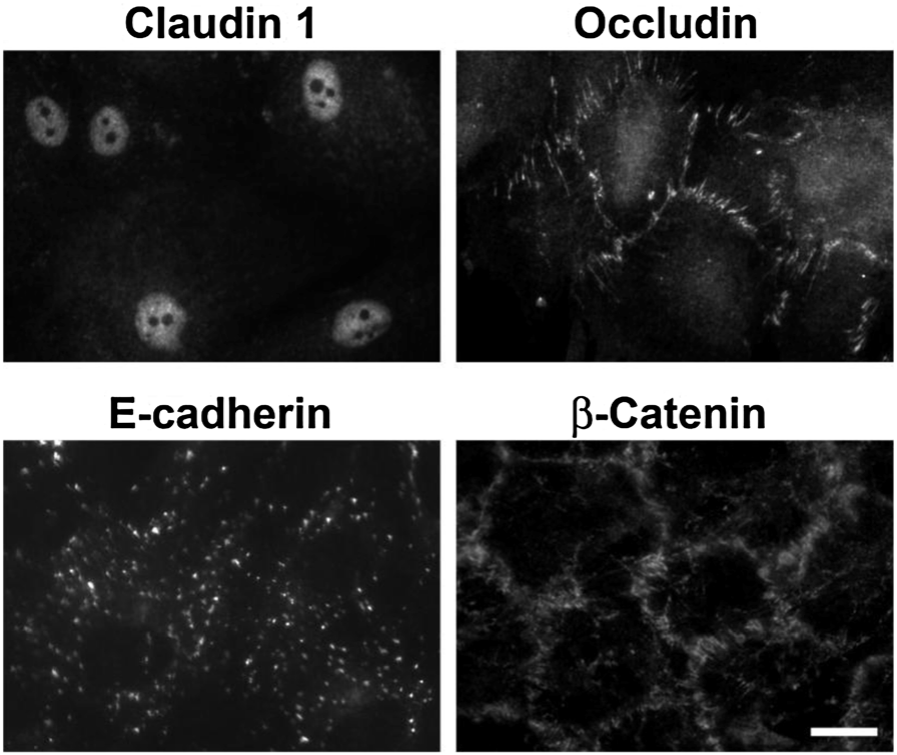
**Immunocytochemical analysis of expression of junctional proteins in CPC-2 cell line derived from human CP carcinoma. **This image shows that CPC-2 cells expressed TJ proteins, occludin and claudin-1 and adherent junctions proteins E-cadherin and β-catenin. Occludin and β-catenin immunoreactivity was largely restricted to the cell-to-cell contact areas, while claudin 1 was found to be restricted to the nuclei of these epithelial cells. Expression of E-cadherin was slim, but largely restricted to cell-to-cell contacts. These findings could be caused by dedifferentiation of CPC-2 cells, when compared to pathologically unaltered CPE. Scale bar = 10 μm. Reproduced with permission from the author [27] .

These cells have been used for several studies. Usually, cells were seeded at a density of 10^4^ cells/cm^2^ (Dr. Joanna Szmydynger-Chodobska, Brown University, USA, personal communication) on collagen-1 coated plastic in high-glucose DMEM supplemented with 10% FCS. These cells are slow-growing; their doubling time is 7–10 days (Dr. Joanna Szmydynger-Chodobska, personal communication); depending on initial seeding density, it may take 2–3 weeks for them to reach 90% confluence. They form monolayers that are less regular and consist of larger cells, when compared to CPE cultures of animal origin [27]. They expressed TJ proteins, occludin and claudin-1. Although occludin immunoreactivity was mainly restricted to the TJ areas, claudin 1 was found to be restricted to the nuclei of these epithelial cells (Figure 3). Overall, the staining patterns for these proteins were often irregular. Under phase-contrast microscopy, they often did not show a typical cobblestone-like appearance. Thus, it is unlikely that this cell line could be used to explore the barrier properties of the CPE. However, CPC-2 cells produce endothelin 1 [28] and adrenomedullin [29], which are characteristics of the CPE *in situ*.

#### Human choroid plexus epithelium primary culture

Recently, human choroid plexus epithelial cells became commercially available from ScienCell laboratories (Carlsbad, CA, USA). These are offered in batches of 50,000 cells in a ready-to-use seeding medium; the manufacturer guarantees the ability to further expand for 15 population doublings under specific conditions. It is recommended to seed these cells on poly-L-lysine treated plastic, without specific coating with basal lamina proteins, with a seeding density of 5,000 cells/cm^2^. Cells should be left undisturbed for at least 16 hours, then Epithelial Cell Medium supplemented with 10% FCS, antibiotics, epidermal growth factor and insulin should be added; all of these reagents are available from the supplier of this cell line. However, in some studies, these cells were grown successfully in the medium that included 2% FCS [30]. When cells attach they display polygonal shaped sheets of neighboring cells. The doubling time is 48-72 h. Cells were positive for the epithelial markers cytokeratin-18, -19 and vimentin. Once cells in culture reach 90% confluence, they should be passaged to poly-L-lysine treated plastic (either wells or filters) with the same seeding density. These cells could be passaged up to 15 times, however, some research groups limit their use to 1–4 passages [30], because some of their features change in later passages.

These cells were successfully used in studies that did not require barrier properties of the cellular monolayer: to explore the importance of epithelial V-like antigen in adhesion of CD4+ T lymphocytes to human choroid plexus epithelial cells *in vitro*[30]; to reveal the expression profile of esophageal cancer-related gene-4 in human CP [31] and to explore cellular uptake (rather than transendothelial transfer) of glutaric acid [32]. However, there are several unresolved issues regarding these cells: first, it is not clear what was their origin - whether it was human CPE taken post-mortem or CPs from fetuses after abortions; attempts to obtain this information from the manufacturer were unsuccessful. Second, it was suggested by the manufacturer that hCPE primary cells could be passaged up to 15 times. Taking into account that other primary CPE cell lines have a rather limited life span and that a dedifferentiation and fibroblast contamination is obvious even after 1–2 passages, as in the case of primary cultures of rat [33] or sheep [34] CPE, this raises a concern regarding whether these cells are primary in their nature. Third, images that are provided by the manufacturer (available at http://www.sciencellonline.com/site/productInformation.php?keyword=1310) do not reveal a typical “cobblestone” -like appearance, a hallmark of differentiated epithelial cells in culture.

## Conclusions

Studies on human CP have been performed either on samples taken post-mortem or after neurosurgery, or on cell cultures. Currently, no evidence exists which indicates that a primary or immortalized cell culture of pathologically unaltered human CPE is available. Most studies on human CPE in culture have in fact been performed on malignant CPE, with rather advanced anaplastic features. Thus, a careful interpretation of acquired data is essential for achieving the correct conclusions, since it is clear that findings obtained from these cell cultures cannot be directly extrapolated to the *in vivo* situation. However, emerging data from the HIBCPP cell line indicate that it could be used to produce monolayers that display some the barrier properties, thus this neoplastic cell line might be used to explore transport and other vectorial processes across the CPE. Other available CP cultures could be used to explore processes that do not require the presentation of barrier function. Thus, it appears that studies on human CPE in culture will be successful if the characteristics of a particular cell culture are considered carefully against the aims of the particular study. At the end, a question arises whether studies on human CPE are essential to explore human CP function, or data from animal studies could be extrapolated to humans. With the data published so far, it appears to be rather difficult to give a simple answer to this question and it probably largely depends on the process studied. In example, as mentioned above, it has been shown that the pattern of distribution of aquaporin-1, Na^+^-K^+^-ATPase α1-subunit and Na^+^-K^+^-2Cl^−^ cotransporter in human CPE were very similar to that observed in rat or mouse [11], indicating essential role of these proteins in CSF secretion, which is a fundamental CP function. On the other hand, an *in vitro* study on human CP samples taken during neurosurgery has indicated that nucleoside uptake by the apical CSF facing side of the CPE was most likely mediated by both human equilibrative nucleoside transporter 2 and human concentrative nucleoside transporter 3 and transcript for the latter transporter was abundant [14], while in the rat CPE in primary culture rat concentrative nucleoside transporter 3 transcript was absent [35], which could indicate differences in roles of CPs in humans and rat in nucleoside homeostasis in the brain.

## Abbreviations

ABCB1: ATP-binding cassette sub-family B member 1, also known as P-glycoprotein; ABCB4: ATP-binding cassette, sub-family B, member 4, also known as MDR3; ABCC1: Multidrug resistance-associated protein 1; BCSFB: Blood-cerebrospinal fluid barriers; CP: Choroid plexus; CPC: CP carcinoma; CPE: CP epithelium; CRL: Crown-rump length; CSF: Cerebrospinal fluid; DMEM: Dulbecco’s Modified Eagle Medium; ECF: Extracellular fluid; FCS: Fetal calf serum; HBSS: Hank’s Buffered Salt Solution; hCNTs: Human concentrative nucleoside transporters; hENTs: Human equilibrative nucleoside transporters; HIBCPP: Human malignant choroid plexus papilloma cell line; TEER: Transendothelial electrical resistance; TJ: Tight junctions.

## Competing interests

The author declares that he has no competing interests.

## Authors’ contributions

ZR - sole author. The author has read and approved the final version of the manuscript.
